# Comprehensive Overview of Methods of Pregnancy Termination in Macaques and Marmosets

**DOI:** 10.3390/vetsci11110527

**Published:** 2024-10-30

**Authors:** Tommaso Virgilio, Remco A. Nederlof, Mallory G. Brown, Jaco Bakker

**Affiliations:** 1 Institute for Research in Biomedicine, Università della Svizzera Italiana, 6500 Bellinzona, Switzerland; tommaso.virgilio@irb.usi.ch; 2 Independent Researcher, 2861 XZ Bergambacht, The Netherlands; remco.a.nederlof@gmail.com; 3 Independent Researcher, Baltimore, MD 21224, USA; mallorybrowndaclam@gmail.com; 4 Animal Science Department, Biomedical Primate Research Centre, 2288 GJ Rijswijk, The Netherlands

**Keywords:** marmosets, macaques, abortion, fetal demise, misoprostol, primates, mifepristone, pregnancy termination, cloprostenol, pregnancy

## Abstract

Limited information is available concerning the termination of pregnancy in non-human primates. Thus, a comprehensive review of this topic will be beneficial for veterinary staff in laboratories, zoos, and wildlife rehabilitation centers. The most relevant studies concerning the termination of viable and non-viable pregnancy in non-human primates were analyzed, and dosages, administration routes, adverse effects, and the efficacy of the drugs used are reported. The literature revealed that termination of pregnancy is most commonly performed in marmosets and macaques. The combination of mifepristone and misoprostol was reported to be effective and fast-acting in terminating first-trimester pregnancy in macaques, while cloprostenol was identified as the best agent for use in marmosets. This review also provides insights about the limitations of previously described methods of pregnancy termination and discusses potential alternatives and areas for future investigation.

## Simple Summary

Different strategies for the termination of pregnancy in humans have proven to be efficient and safe. However, few studies have investigated the utility of these regimens in non-human primates. Therefore, this review discusses the most relevant research reporting the termination of viable and non-viable pregnancies in primates. An overview of the clinically applicable drugs is presented, including dosage, administration route, safety, and efficacy.

## 1. Introduction

An abortion is a medical procedure to terminate a viable or non-viable pregnancy. Pregnancy may be terminated for several reasons, including if there is an unwanted pregnancy, if there is fetal death, or if it is medically indicated. Several methods, including pharmaceutical termination, manual vacuum aspiration, dilation and evacuation, and curettage, are used to perform pregnancy termination depending on weeks’ gestation, provider skill, availability of appropriate sedation or anesthesia, costs, clinical setting, and state policies and regulations [1,2,3,4,5,6]. All these approaches are efficient and safe, but physical methods are more invasive than pharmaceutical methods. Consequently, pharmaceutical pregnancy termination is the preferred method whenever applicable [1,2,3,6].

Standard pharmaceutical induction of parturition is performed using prostaglandins (PGs), antiprogestins, or synthetic estrogen antagonists. Generally, a regimen that combines multiple drugs is more effective than single agents due to the drugs’ synergism or the need for lower individual doses when multiple drugs are combined, thus reducing the risk of each agent’s adverse effects [2,3,6,7,8].

Despite the abundance of studies demonstrating the safety and efficacy of several pharmaceutical and physical methods of medical pregnancy termination in humans [3,9], few studies have investigated the utility of these methods in non-human primates (NHPs). In fact, very limited information is available concerning the medical termination of non-viable and viable pregnancies in NHPs. Additionally, a common problem for medical intervention is that few pharmaceutical products are officially approved for NHPs, resulting in the off-label use of drugs [10]. Therefore, a comprehensive overview of the published methods for termination of pregnancy in NHPs, including dosage, administration route, safety, and effectiveness, will provide strategies to refine veterinary health care procedures in laboratories, zoos, and rehabilitation wildlife centers working with NHPs. It will also highlight areas for future investigation of possible alternative methods.

To identify all the relevant literature, we conducted a search for books, book chapters, peer-reviewed publications, conference abstracts, and newsletters in academic literature databases, including PubMed, Google Scholar, EMBASE, CINAHL, POPLINE, Cochrane Library, and Global Index Medicus. We excluded toxicity studies (e.g., lead and triacetyl-6-azauridine administration) from our review because the termination of pregnancy was an accidental, secondary effect of a substance, making its intentional use to induce parturition an off-label, unauthorized procedure [11,12,13,14]. For the same reason, we excluded different classes of antibiotics, such as macrolides, quinolones, tetracyclines, sulfonamides, and metronidazole, which are associated with an increased risk of miscarriage during first-trimester pregnancy in women [15,16,17].

## 2. Physiology of Pregnancy Maintenance

The primate species most intensively studied with respect to medical termination of pregnancy include the genus *Macaca*, most commonly the rhesus (*Macaca mulatta*) and cynomolgus macaque (*Macaca fascicularis*), and the common marmoset (*Callithrix jacchus*).

Similar to humans, macaques have an approximately 30-day menstrual cycle with menstrual bleeding. However, rhesus macaques are seasonal breeders, while cynomolgus macaques mate and give birth throughout the year. In both species of macaques, pregnancy ranges from 133 to 182 days, with 80% of births occurring between 160 and 175 days and an average gestation length of 165 days [18,19,20]. A standard pregnancy trimester length in macaques is approximately 55 days; the first trimester is defined as gestational days 0–55, the second trimester as days 56–110, and the third trimester as days 110–165 [21]. The macaque placenta is chorioallantoic, discoid, villous, deciduate, and hemochorial, meaning fetal trophoblasts erode through the maternal endothelium and directly contact the maternal blood. During early development, the blastocyst superficially adheres to the uterine wall opposite the site of initial attachment. This eventually forms the secondary placental disc, creating the bidiscoid placenta pattern seen in approximately 80% of macaque pregnancies [22].

Pregnancy is maintained by progesterone, which is produced initially by the corpus luteum and subsequently by the placenta. The luteal–placental transition of progesterone secretion occurs around day 28, which corresponds to the end of a macaque’s normal fertile ovulatory cycle; by day 35, the presence of the ovary is no longer required for successful maintenance of gestation [23,24,25,26]. Female macaques enter menopause, or reproductive senescence, at approximately 25 years old. This corresponds to a decline in sex hormone production, a discontinuation of ovulation, and an end to reproductive capability [27,28,29,30].

Common marmosets are cooperative breeders that exhibit alloparenting, meaning both the male and female care for the infants [31]. Marmosets typically give birth to fraternal twins, but triplets and quadruplets are common in captivity. The ovarian cycle is approximately 28 days in marmosets, and, unlike humans and macaques, they do not menstruate, and they exhibit a fertile post-partum estrus [31]. The average gestation period is 144 days [31]. Marmoset placentas differ significantly from those in macaques, which contributes to many of the unique reproductive attributes seen in these primates. The placenta grows rapidly from gestational days 0 to 100, and, early in gestation, each embryo’s placental unit fuses and anastomoses. This allows the exchange of precursor stem cells among embryos, resulting in chimeras. The naturally occurring chimerism of marmoset offspring is a unique feature of this species that offers many advantages as a research animal model [32,33].

Although marmosets do not menstruate, the patterns of their reproductive hormones, especially chorionic gonadotropin, are very similar to humans. Ovulation occurs about 24 h after the luteinizing hormone surge, corresponding to 36 h after the midcycle peak in estradiol levels. Implantation occurs on days 9 to 10 after ovulation, and the corpus luteum is necessary to sustain pregnancy for nearly 48 days after ovulation. The luteal–placental shift of progesterone production occurs around gestational day 40 [34,35,36,37]. Although there is evidence of ovarian aging in marmosets, they do not experience menopause or undergo the same age-related biological changes seen in humans and macaques [38].

For all primates, pregnancy maintenance requires that the dam’s body does not immunologically reject the fetus, which differs antigenically from the dam due to paternal DNA, but also that the dam’s immune system is robust enough to protect against pathogen transmission to the fetus. To allow for this nuanced relationship, the maternal–fetal interface of endometrial cells and placental trophoblasts contains a myriad of immune and signaling cells, and there are often redundant systems to ensure proper development [39]. For example, during blastocyst implantation in marmosets, both the hormone relaxin and its receptor, RXFP1, are upregulated, and macrophages infiltrate the uterine wall. These redundant changes ensure immunotolerance of the fetus and successful implantation in the uterine wall [40]. Since marmoset fetuses’ placentas anastomose and fetal blood encounters allogenic tissues and antigens, marmosets require stronger immunosuppression during pregnancy than humans whose placentas do not anastomose with twin fetuses [41].

## 3. Indications for Termination of Pregnancy

Indications for termination of a pregnancy include accidental, undesired breeding and pregnancy in animals intended for research. Usually, animals not destined for breeding are treated with contraceptive agents. Unfortunately, contraceptive failure may occur for a variety of reasons in NHPs, making undesired pregnancies relatively common in colonies hosting large numbers of animals [42]. Additional reasons to terminate a pregnancy include clinical diseases that put the life of the mother at risk (e.g., pregnancy toxemia) and abnormal pregnancies (i.e., congenital defects, fetal mummification, and fetal demise) [43,44,45,46,47,48,49,50,51]. The nature of these cases is accidental. However, intentional termination of pregnancy is also performed. For example, elective pharmaceutical termination of pregnancies and cesarean deliveries of NHP infants are often performed as a scheduled part of experimental protocols near the completion of gestation [23,52,53,54,55].

## 4. Pharmaceutical Methods

Administration of one or more pharmaceutical drugs, alone or in combination, can induce parturition or terminate pregnancy. The most efficient strategy to ensure abortion is to administer two drugs with different pharmacokinetics. The first drug blocks progesterone production or action, resulting in a thinning of the uterine lining and disrupting the body’s signal to maintain pregnancy. The second drug induces the body to expel the embryo or fetus [1,3]. The abortifacient drugs that are used in NHPs are listed in Table 1. Currently, in human medicine, combined regimens of drugs are more effective than single agents [1,2,3,6,7].

### 4.1. Prostaglandins and Combinations

Prostaglandins (PGs) are physiologically active lipid autacoids formed during arachidonic acid metabolism [56]. These compounds are divided into several classes; some have stimulatory effects on the myometrium, thus inducing uterine contractions, while others degrade the corpus luteum and prevent the ovary from producing progesterone to maintain pregnancy [57]. Consequently, PGs represent one of the most common medical treatments to induce fetal expulsion and termination of pregnancy [58]. Alprostadil (PGE1), dinoprostone (PGE2), and dinoprost (PGF2α), or their synthetic analogs (misoprostol, carboprost, cloprostenol, or sulprostone), are used worldwide for termination of pregnancy in women [3,58]. Unfortunately, reports of their use in NHPs are relatively scarce.

Injection of PGF2α into the amniotic sac in three second-trimester-pregnant rhesus macaques successfully terminated pregnancy with minimal adverse effects [59]. Pregnancy was terminated between 12 and 18 h for the macaque receiving 5 mg (*n* = 1) and between 36 and 48 h for the macaques receiving 1 mg (*n* = 2). Despite the low sample size, the absence of adverse reactions in this study suggests an advantage to amniotic sac injection over other conventional routes, such as intravenous (IV) infusion. Diarrhea, vomiting, and local cutaneous inflammatory reactions at the site of injection are common adverse reactions associated with IV administration. In humans, the incidence of secondary adverse events also correlates with the PG class used and the dose and duration of therapy [60,61].

Supporting the importance of the route of administration, another study reported that IM injection of single PGs to pregnant rhesus macaques was not effective in terminating first-trimester pregnancy [62]. IM injections of a PGF analog alone for two days (7.5 mg every 12 h) or for three days (7.5 mg every 8 h) terminated pregnancy in only one of three and one of two macaques, respectively. Similarly, a PGE analog (0.5 mg IM every 8 h) and PGF2α (3 mg every 12 h for two administrations) showed low efficacy in terminating pregnancy. Macaques undergo a luteal–placental shift in progesterone production around day 28 post-fertilization, and by day 35, the corpus luteum is no longer required to maintain pregnancy. This luteal–placental shift may explain the poor abortifacient efficacy of single PG administration in these species. Once the shift occurs, the placenta produces progesterone to maintain pregnancy rather than the corpus luteum. If a PG’s mechanism of action induces luteolysis of the corpus luteum after the shift has occurred, it will not terminate the pregnancy. Thus, a combination of PGs with both ovarian and uterine sites of action might be more effective for interrupting first-trimester pregnancy. To test this theory, the same authors gave IM PGF analog (7.5 mg every 8 h for three administrations) combined with IM PGE2 analog (0.5 mg for three administrations) to three macaques on day 28 of their fertile menstrual [62,63]. All three first-trimester macaques successfully aborted. Additionally, the combination of a PGF analog (7.5 mg every 12 h for two administrations or 5 mg every 8 h for three administrations) with PGF2α (3 mg twice or 1 mg three times) terminated first-trimester pregnancy in six macaques. However, this regimen was no longer efficient when administered in the third trimester of pregnancy [62,63]. Although the number of animals treated in these studies was low, the results indicate that combinations of synthetic PGs with both ovarian and uterine sites of action represent a potential option for interrupting pregnancy during the first trimester in rhesus macaques due to the luteal–placental shift in progesterone production.

One report described the effects of IM PGF2α in rhesus macaques (*n* = 5) during the third trimester of pregnancy and the peripartum period [64]. Animals were given 10–15 mg/day of PGF2α, beginning on days 148–151 and continuing until delivery. The daily maternal injections induced abortion within 2–6 days in all macaques. The fetuses were either stillborn or were found dead in their cages on the morning following delivery. Because these fetuses were not macerated, they were believed to have died intrapartum. Pre-treatment with estradiol, which is necessary for normal parturition, did not influence the results (*n* = 2).

The combination of PGF analogs with mestranol, a synthetic estradiol, has also been investigated in rhesus macaques (*n* = 12). A study showed effective termination of pregnancy in 92% of treated macaques (11 out of 12) when the combination of PGF analog and mestranol was administered on day 28 of fertile menstrual cycles, corresponding to the first trimester of pregnancy [63]. However, adverse reactions were observed in both pregnant and nonpregnant control macaques, including anorexia (44%), diarrhea (16%), and emesis (5%) [63]. Mestranol combined with low-dose 15M-PGF2α terminated pregnancy in only 40% of macaques (two out of five), showing no advantage compared to 15M-PGF2α alone [65]. It is hypothesized that mestranol impairs the steroidogenic potential of the corpus luteum in the first trimester of pregnancy but is not sufficiently active to terminate gestation alone [65].

Another study compared SC and IV administration of PGE2 or PGF2α to rhesus macaques between days 30 and 127 of pregnancy [66]. When given SC (15 mg for 1–5 consecutive days), pregnancy was terminated in 55% of macaques (six out of eleven) injected with PGF2α prior to day 41 and in 100% of macaques (two out of two) injected after day 100. Pregnancy was not terminated in any of the four animals injected SC between days 41 and 100. PGE2 was effective in only one of two animals injected SC in the first trimester of pregnancy. PGF2α, administered IV at a dose of 45 mg, terminated pregnancy in 43% of macaques (three out of seven) treated prior to day 40 of pregnancy. All IV infusions were administered on two or three consecutive days for a period of four or eight hours each day. When administered before 40 days gestation, both SC and IV routes of both PG drugs caused severe bloody vaginal discharge during pregnancy termination. When the drugs were administered later in gestation, the fetus and placenta were expelled intact with no complications.

The abortifacient effect of synthetic PGE 15(S) 15-methyl-prostaglandin E2 (mPGE2) was studied in five pregnant pigtailed macaques (*Macaca nemestrina*) between 70 and 135 days [67]. Initially, 2 mg of mPGE2 was administered IM every 2–4 h, but this dose caused severe uterine contractions and abdominal pain. To simulate more physiologic contractions, the dose was decreased to 0.5–1 mg. Despite its effectiveness in inducing abortions in humans, the lower doses of IM mPGE2 did not reliably produce abortion in macaques.

PG therapy has been studied and refined for many decades. The sections below provide an overview of specific PGs that are notable because of their efficiency, mechanism of action, or common use.

#### 4.1.1. Misoprostol

Misoprostol is a synthetic PGE1 analog of primary importance, mainly due to its common use in combination with the antiprogestin mifepristone (see Section 4.2.2). While some studies report its individual use in humans [68,69], the authors found no reports of using misoprostol alone in NHPs. In the first trimester of pregnancy, misoprostol was successful in terminating pregnancy in women. Moistening the tablets before intravaginal insertion does not seem to improve the efficacy of intravaginally administered misoprostol for terminating pregnancy [70,71]. Common adverse effects include uterine cramping, nausea, vomiting, fever, chills, and diarrhea. Adverse effects occur frequently in single-agent misoprostol administration in humans, but they are rare in combination with mifepristone [68,69,72,73,74]. This could be due to synergism between the drugs or to the lower dose of misoprostol required when combined with mifepristone. For this reason, combining misoprostol with mifepristone is more common than single-drug regimens.

#### 4.1.2. Cloprostenol

Cloprostenol is a synthetic PGF2α analog with a unique mechanism of action: it induces lysis of the corpus luteum while inhibiting progesterone, estradiol, and inhibin A synthesis and release [35,75].

Female common marmosets were treated with IM cloprostenol between days 1 and 64 after ovulation at doses ranging from 1.2 to 50 μg/kg of body weight. The treatment of 131 pregnant animals resulted in an 87% luteolysis rate with no adverse reactions reported in any animal [76]. The luteolytic effect was independent of the dose. Luteolysis was observed when cloprostenol was administered after day 5 post-ovulation, but it failed to induce luteolysis during the early luteal phase (days 1–4) when the corpus luteum was immature.

In another study, seven pregnant common marmosets were administered a single IM injection of 0.5 μg of cloprostenol [75]. This induced luteolysis between days 19 and 43 of pregnancy. Importantly, there were no adverse reactions, and marmosets conceived immediately following the cessation of the treatment.

The mechanism of action of cloprostenol is hypothesized to have a direct effect on the corpus luteum rather than on uterine contractility. Cloprostenol may act by inhibiting luteinizing hormone or human chorionic gonadotropin, both of which are required by the corpus luteum to produce progesterone and maintain pregnancy [77].

These studies highlight several advantages of administering cloprostenol to marmosets, including its use as a sole agent, its high efficiency, and the absence of side effects. Its use has not been investigated in macaques, which could be an area of future research.

#### 4.1.3. Dinoprostone

Dinoprostone is a natural PGE2 analog used as an abortifacient and labor induction agent in women [78,79]. However, there is only one report of its use in NHPs [80]. The case involved a 4-year-old, 600 g, nulliparous female golden lion tamarin (*Leontopithecus rosalia*) that presented weak and tachypneic with a fetal head and shoulders protruding from the vaginal orifice. The animal had a history of melengestrol acetate implantation at 1 year of age, which was removed ten months prior for breeding purposes. The fetus was diagnosed as non-viable and was delivered using lubricant and gentle digital manipulation; however, the placenta was retained. IM administration of dinoprost, starting at 0.015 mg and increasing every 12 h over 48 h to a final dose of 0.12 mg, was unable to induce placental expulsion. On the fifth day, as the clinical condition of the tamarin worsened, an ovariohysterectomy was performed to remove the retained placenta. In this case, dinoprostone was ineffective, possibly due to an insufficient dosage. While a single case report does not provide conclusive evidence of the efficacy of dinoprostone in NHPs, future research could investigate alternative dosing regimens or combining dinoprostone with other PGs or antiprogestins.

#### 4.1.4. Carboprost

Carboprost, a 15-methylated analog of PGF2α, injected IM in 815 women, had a 78% rate of complete abortions, an 18% rate of incomplete abortions, and an overall success rate of 96% [81]. The most common adverse reactions, including vomiting and diarrhea, are generally well tolerated by women. Discontinuation of the treatment due to adverse effects is, therefore, rarely required [82].

The ease of administration and potential for self-administration in women have prompted investigation into developing a vaginal delivery system for PGF2α. Using rhesus macaques, researchers explored the biological activity of silicone vaginal rings containing 15(S)-15-methyl PGF2α methyl ester [83,84]. Five first-trimester rhesus macaques (29 to 35 days post-mating) were treated with vaginal rings left in place for 48 h. Vaginal bleeding occurred in all animals within one day due to the positioning of the rings, and pregnancy was terminated in 80% of macaques (four out of five).

A cohort of three second-trimester rhesus macaques (83 to 118 days of gestation) had vaginal rings in place for 5.5 to 8 h. Cervical dilation and vaginal bleeding were observed within four to five hours of insertion. All macaques aborted within 24 to 48 h. Moderate vaginal bleeding continued for two to eight days post-abortion. These results demonstrate that silicone vaginal rings containing 15(S)-15-methyl PGF2α methyl ester are a valuable, low-invasive option to induce luteolysis, uterine contractions, and abortion in rhesus macaques [83].

Mestranol, a synthetic estradiol, is synergistic with 15-MPGF2α and PGF1α in terminating first-trimester pregnancy when injected at day 28 of the fertile menstrual cycle of macaques [63].

### 4.2. Antiprogestins

Antiprogestins are competitive progesterone antagonists acting on both progesterone (P4) and glucocorticoid receptors [85]. Their abortifacient mechanism relies on their capacity to block the progesterone necessary to sustain pregnancy and fetal development, thus leading to pregnancy interruption. Importantly, antiprogestins are often administered in combination with PGs to facilitate the expulsion of the dead fetus. Antiprogestins are the most common pharmaceutical method to terminate pregnancy in humans, and the different agents are reviewed individually here.

#### 4.2.1. Mifepristone (RU-486)

Mifepristone, also known by its developmental code name RU-486, is a synthetic steroid compound that affects human fertility through its antiprogesterone and antiglucocorticosteroid activity [86,87]. Multiple groups have studied the contraceptive and abortifacient effects of mifepristone in macaques [24,88,89,90,91,92,93,94,95].

An early study of mifepristone in NHPs investigated the appropriate dose range of RU-486 for terminating pregnancy before and after the luteal–placental shift in cynomolgus macaques during the first trimester of pregnancy [24]. RU-486 was injected IM at doses of 1.0, 2.5,12.5, or 25.0 mg/kg from gestational days 15 to 18 (Group 1; *n* = 11) or from gestational days 26 to 29 (Group 2; *n* = 9). In Group 1, pregnancy loss was observed in 90.9% of the cases (10 out of 11), reporting seven abortions during gestational day 15–20, one on gestational day 56, two early embryonic deaths with retained gestational sacs, and one maintained pregnancy. Similarly, pregnancy loss in Group 2 was observed in 88.9% of cases (eight out of nine), with eight abortions between gestational days 26 to 29 and one maintained pregnancy. The authors of this study suggested that RU-486 may be more effective after the luteal–placental shift due to the higher rate of complete abortions and more consistent outcome in Group 2. Notably, one fetus exposed to mifepristone (2.5 mg/kg) during gestational days 15–18 developed craniofacial malformations. Although it is unclear whether these abnormalities were spontaneous or directly related to mifepristone exposure, a treatment-related adverse effect cannot be excluded.

Subsequent studies of mifepristone in macaques presented conflicting results. Investigating the most efficacious route of administration for 20 mg mifepristone in cynomolgus macaques, one group reported that pregnancy termination was low with oral administration (17% termination, *n* = 6) but high with IM injection (83% termination, *n* = 52) [96]. In another study, three third-trimester-pregnant macaques (species not reported) received IM mifepristone at 10 mg/kg [97]. Despite a significant decrease in amniotic fluid volume within 24 h, this protocol did not induce abortion in any animal. These results conflict with the earlier proposed increased efficacy of mifepristone after the luteal–placental shift in cynomolgus macaques.

The abortifacient efficacy of mifepristone has been compared to that of the antiprogestin HRP 2000 in first-trimester-pregnant cynomolgus macaques [92]. Animals were treated on gestational days 23–26 either orally with a 0.5 or 5.0 mg/kg dose for both drugs (*n* = 5 per dose, per drug) or IM at a dose of 0.5 mg/kg (*n* = 5 per drug). High rates of abortion were observed with IM administration of both drugs (three out of five animals for mifepristone; four out of five animals for HRP 2000) and with the 5.0 mg/kg oral dose of mifepristone (four out of five animals). In contrast, the lower oral dose (0.5 mg/kg) induced abortion only in 2 out of 5 cases for mifepristone and in 0 out of 5 cases for HRP 2000. This study indicates that both HRP 2000 and mifepristone are effective in terminating first-trimester pregnancy when given IM but that only a high oral dose of mifepristone is effective at terminating pregnancy.

#### 4.2.2. Mifepristone Combined with Misoprostol

The combination of mifepristone and misoprostol is considered the most effective, common, and fast regimen in women who want to abort [3]. A standard administration protocol in women consists of a single oral dose of 200 mg of mifepristone followed by 800 µg of buccal, sublingual, or vaginal misoprostol 24 to 48 h later [3,98,99,100,101,102,103,104].

Multiple publications describe the efficacy of mifepristone and misoprostol in macaques. One study administered mifepristone (1 mg/kg SC) and misoprostol (1 mg/kg in the posterior fornix of the vagina) to six pregnant rhesus macaques at various stages of pregnancy [55]. All macaques aborted within three days, and no adverse reactions were observed. When given as the sole agent, mifepristone was associated with dystocia and fetal suffocation in utero before expulsion. It was hypothesized that this drug may not loosen the birth canal, leading to these adverse effects. The addition of misoprostol to the pharmaceutical regimen resolved all complications.

Another study aimed to optimize the administration protocol of mifepristone and misoprostol in pregnant cynomolgus macaques [96]. Misoprostol was given either immediately or 24–48 h after 20 mg IM mifepristone. Misoprostol was administered either orally or buccally at dosages of 200 or 400 µg. The rate of successful pregnancy termination did not differ if misoprostol was delivered via the buccal or oral route; however, the buccal route was associated with lower rates of retained fetal tissue. The authors determined the optimal protocol as 20 mg IM mifepristone resuspended in Captex^®^ oil followed by 200 µg misoprostol crushed and placed in the cheek pouch 48 h later. Ultrasonic verification of fetal viability is recommended one week following the treatment with continued ultrasonic monitoring every two to four weeks until the uterus is completely closed and no fluid or retained fetal tissues are observed in the uterine cavity. In case of unsuccessful therapy, the authors recommend injecting methotrexate (50 mg/m^2^) directly into the fetus under ultrasound guidance and confirming fetal demise one week later.

#### 4.2.3. Lilopristone (ZK 98.734)

The antiprogestin lilopristone, also known by its developmental code name ZK 98.734, is structurally related to mifepristone. Its relative binding affinity for progesterone receptors in the human uterus is like that of mifepristone, but its antiglucocorticoid activity is lower [105], and it is suggested to be more potent as an abortifacient than mifepristone in laboratory animals [106].

In its first reported use in NHPs, lilopristone was administered at a dose of 5 mg IM for three consecutive days to first-trimester-pregnant marmosets (*n* = 7) on days 24–26 of the mid-cycle estradiol peak [107,108]. The treatment successfully terminated gestation in all animals, and they had ovulatory post-treatment cycles of normal duration. Lilopristone exhibited specific binding to the myometrial cytosol fraction and displaced the binding of 3H-progesterone to progesterone receptors. Thus, the abortifacient effect of lilopristone was speculated to result from either its luteolytic action or its ability to block progesterone receptors in target tissues.

A follow-up study was conducted to assess the effects of lilopristone at four stages of gestation, using a 5 mg IM dose [109]. The treatment was initiated relative to the mid-cycle estradiol peak (day 0) as follows: group 1 (*n* = 8) received lilopristone from days 10 to 12 (around the time of implantation); group 2 (*n* = 5) from days 20 to 22; group 3 (*n* = 5) on day 40 (before the luteoplacental shift in progesterone synthesis); and group 4 (*n* = 7) on day 80 (after the luteoplacental shift). Pregnancy was terminated in all marmosets from group 1. In groups 2 and 3, vaginal bleeding occurred between 32 and 46 h after treatment initiation, all the pregnancies terminated, and three marmosets in group 3 showed signs of conceptus resorption, as indicated by abdominal palpation. In group 4, lilopristone induced abortion in all marmosets, but the fetuses were expelled between 20 and 48 h after treatment began. Supporting the previous evidence, this study demonstrated that lilopristone is a potent abortifacient in common marmosets.

The same research group investigated the effect of 25 mg lilopristone administrated SC, once daily, as a contraceptive and abortifacient in female bonnet monkeys (*Macaca radiata)* (*n* = 14) [108,110,111]. Ten monkeys received lilopristone from day 30 to day 33 from the last menstrual cycle, while four macaques were treated between days 60 and 63. The treatment induced abortion in 80% of animals (eight out of ten) on days 30–32 of the menstrual cycle. In the other two macaques, the pregnancy was unaffected and remained viable. One animal was treated with lilopristone for a second time on days 65–67, and the fetus was expelled on day 67. The second animal was allowed to reach full-term pregnancy, and a normal fetus was delivered on day 175. In the group treated between days 60 and 63, all four animals aborted within six days after the initiation of treatment. These results indicate that lilopristone may be a good abortifacient in macaques, depending on the gestational time of administration, but the small sample sizes may affect the interpretation of the results. Future studies with larger sample sizes should investigate this therapy in macaques.

### 4.3. Methotrexate

Methotrexate is a chemotherapeutic agent that halts DNA synthesis through inhibition of dihydrofolate reductase; as a result, it is toxic to the fetus when injected directly into the trophoblast [112,113]. This agent has infrequently been used in humans to terminate pregnancy when mifepristone is unavailable [114,115,116] or, more commonly, to interrupt ectopic pregnancy [117].

In first-trimester-pregnant cynomolgus macaques (*n* = 15), intrafetal injection of 50 mg/m^2^ methotrexate had a 100% efficacy in terminating pregnancy [95]. In this study, methotrexate induced a higher frequency of pain, diarrhea, vomiting, hyporexia, and dehydration than mifepristone. Moreover, it was associated with retention of fetal tissue in utero (14 out of 15) [96]. Despite the remarkable efficiency in terminating first-trimester pregnancy, there are serious concerns about the administration of methotrexate due to the high frequency of severe adverse reactions and the occupational hazard associated with the use of a chemotherapeutic agent. Thus, its application should be considered only when alternative methods are unavailable [96]. Additionally, to facilitate the expulsion of the dead fetus, misoprostol should be administered seven days after methotrexate injection [118].

An embryotoxicity study was conducted involving first-trimester-pregnant rhesus macaques (*n* = 25). The macaques were treated with methotrexate at various doses (0.5–4.0 mg/kg/day) for different durations (1–24 days) during gestational days 17 to 45 [119]. The authors did not describe the route of administration. The lowest dose of 0.5 mg/kg/day was tolerated for 24 days without adverse reactions. A dose of 1 mg/kg/day was also well tolerated for up to six days. However, when administered for 10 days or longer (*n* = 4), this dose resulted in one maternal death, one abortion (after 19 days), and one severely growth-stunted 100-day fetus with bone abnormalities. One embryo survived for 22 days with this dosage without observed adverse effects, as confirmed by a hysterectomy at 100 days of gestation. A dose of 3.0 mg/kg/day had no effect when given for one to three days, but when eight macaques were treated for four days between gestation days 20 and 45, five aborted and three produced normal fetuses at 100 days. A dose of 4.0 mg/kg/day caused abortion in two out of three macaques after just two days of treatment.

Taken together, these results indicate that methotrexate does not offer high safety or abortifacient reproducibility; therefore, its administration should be limited to cases in which other regimens are not possible, and complementary treatments, such as misoprostol, should be implemented if possible.

### 4.4. Heterologous Placental Immunoglobulins

One study reported using heterologous goat and rabbit anti-rhesus monkey placental immunoglobulin (IgG) to induce abortion in first-trimester-pregnant rhesus macaques (*n* = 9) [120]. Anti-placental IgG binding to the trophoblastic tissue activates the complement system and results in immune-mediated tissue destruction and abortion. A total of 600–1000 mg of mixed goat and rabbit anti-placental IgG were injected IV over three days into macaques between 39 and 54 days of gestation. Abortion occurred in seven of the nine macaques injected with IgG, with two requiring a second administration protocol. No anaphylaxis or adverse reactions were observed, and the authors hypothesized a passive immunization mechanism as the mediator of abortion. This single report may support the use of heterologous IgG to interrupt pregnancy in NHPs. However, the lack of knowledge of the specific placental antigen raises concern about the safety and reproducibility of such an approach, and more research is needed.

## 5. Physical Methods

As an alternative to pharmaceutical methods, there is a range of physical methods that provide more traditional, but often invasive, alternatives for pregnancy termination.

Vacuum aspiration, commonly referred to as suction curettage, removes the fetus and uterine contents by gentle suction. However, transcervical rupture of the fetal membranes for the purpose of inducing second-trimester abortion in women is unreliable and may lead to serious complications such as intrauterine infection [121]. In macaques, transcervical uterine aspiration is not feasible due to their tortuous cervical anatomy [122].

**Figure 1:**
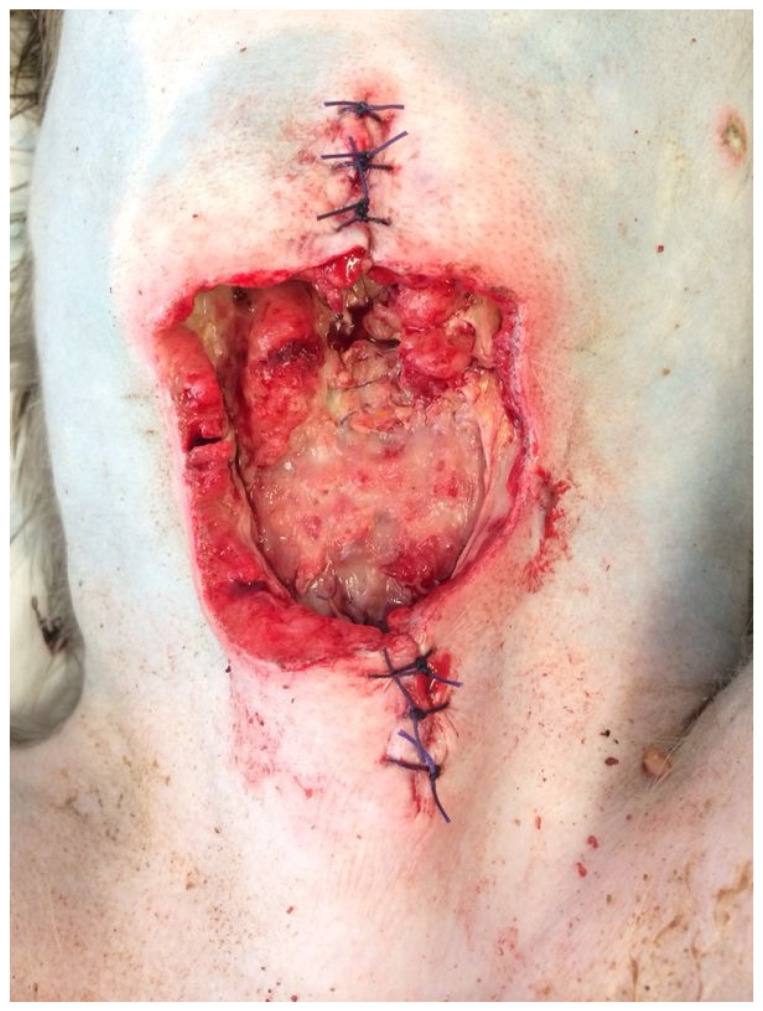
Dehisced abdominal laparotomy incision after cesarean section in a rhesus macaque (photograph provided by Biomedical Primate Research Centre, Rijswijk, The Netherlands).

Hysterotomy, or cesarean delivery, is most commonly performed as an emergency procedure when complications occur during parturition [123,124]. However, elective cesarean sections can also be done to remove the dead fetus in cases of fetal demise and fetal mummification [44,47,48,49,50,51]. In laboratory NHPs, elective cesarean sections are sometimes performed as part of experimental protocols near the completion of gestation [52]. Details of the operative technique are described in detail for macaques [52,125] and marmosets [126]. One risk factor of cesarean delivery is placental retention, which reportedly occurs in 37.0% of baboons and 4.7% of cynomolgus macaques that have had at least one prior cesarean section [127]. Interestingly, the type of uterine incision for a cesarean section in women may correlate with the risk of placental retention in subsequent pregnancies [128]; however, this has not been evaluated in NHPs. An additional complication that occurs in both humans and NHPs is wound dehiscence post-operatively, as seen in Figure 1. Unlike with humans, it is difficult to limit NHP activity while the surgical incision heals and to prevent them from manipulating and removing their sutures once returned to the housing facility. Wound dehiscence can lead to infection and require additional surgeries [129].

Focused ultrasound, a low-invasive alternative method, was explored in six first-trimester pregnant rhesus macaques [130]. During the gestational period of 37 to 66 days, a high-intensity focused ultrasound therapeutic unit exposed the macaques to varying modes of ultrasounds to terminate first-trimester pregnancies. B-mode ultrasound of the gestational sacs showed a significant lethal effect on the fetus. Between two and eight hours after focused ultrasound exposure, all rhesus macaques experienced vaginal bleeding, and five of them presented complete discharge of the gestational tissue between 17 and 164 h after ultrasound exposure. The vaginal discharge was a mixture of dead tissue and blood, and the gestational sac was almost intact upon discharge. Of the six rhesus macaques studied, five underwent complete abortions and one experienced an incomplete abortion with retention of fetal tissue. This animal required surgical removal of the remaining tissues 166 h after focused ultrasound treatment. All macaques resumed their menstrual cycles 50 days after focused ultrasound treatment. Importantly, all six animals became pregnant again through natural mating, starting from six to nine months after the ultrasound intervention. No deformities were observed in the first, second, or third generations after long-term observation. These results suggest that focused ultrasound may be a low-invasive, safe, and effective method for terminating first-trimester pregnancies in macaques.

## 6. Conclusions

This review summarizes the commonly used drugs and methods for pregnancy termination in NHPs, particularly macaques and marmosets. It addresses the lack of recent review articles on this topic, facilitating the sharing of veterinary medical procedures among institutions and providing practitioners with strategic references.

A weakness of several studies reported here is the low number of NHPs used during investigations. This limitation diminishes the relevance of such studies because, in many cases, the results were considered inconclusive due to the absence of statistical and biological significance. Even the reduction principle of laboratory animal experimentation recommends that experimental groups should be formed with the lowest number of animals that still allows for conclusive results based on data variation and the statistical power of the methods used. Thus, most of the protocols described here require more extensive studies to determine optimal use in NHPs.

In addition, most of the studies that we identified in the scientific databases were published 30 to 40 years ago. This is not a limitation per se, but it raises concerns about why there are not more recent reports. A possible explanation is that when individual institutions establish protocols for common veterinary procedures, they are not always shared or considered for publication, which hampers their reproducibility among other institutions and investigators. The paucity of more recent literature might also be due to a lack of novel strategies and innovative solutions. All these factors hinder practitioners who seek to refine or introduce a new medical procedure in the routine clinical veterinary management of a colony of NHPs. Given these obstacles, comprehensive literature reviews such as this one are of particular value.

We have highlighted studies of both macaques and marmosets, as they are two of the most common NHP models. However, it is important to keep in mind the species specificities of reproductive systems. For example, marmosets exhibit higher reproductive efficiency than macaques, with a potential for different sensitivities to reproductive techniques or termination methods [131]. Thus, it is not recommended to simply translate the protocols described for marmosets to macaques, or vice versa, as they might lead to different results, efficiency, and safety. Preliminary validations should always be considered.

It is critical to note that most of the pharmaceutical protocols we have described were only tested in first-trimester pregnancies. One exception is the study that investigated mifepristone use in both first- and third-trimester pregnancy in macaques. This drug was highly effective at inducing abortion in the first trimester but not in the third trimester [24]. Given the substantial changes occurring in the fetus and to the reproductive system during pregnancy, it is inadvisable to draw conclusions about terminating third-trimester pregnancies [24,64] or overdue pregnancies based on studies of first-trimester abortions. More extensive and systematic research is required to identify the best regimens for terminating third-trimester pregnancies in NHPs.

The administration of drugs poses unique obstacles in NHPs, especially in oral treatments, which are often unappealing in smell or taste and are not easily masked in food. Implementing positive reinforcement training can solve some of the oral administration challenges [132]. Nevertheless, many NHP studies overcome this problem by injecting drugs subcutaneously (SC) or intramuscularly (IM), which more reliably ensures the animal receives the full dose. Alternatively, the insertion of silicon vaginal rings releasing abortifacient drugs could provide an alternative to oral or injectable medication, but practitioners should be aware of vaginal bleeding as a potential side effect after insertion [83,84]. Importantly, administration routes are not interchangeable, as the pharmacokinetics, biodistribution, bioavailability, and catabolism of a drug might change depending on the injection method, thus requiring adjustments and validation of the therapeutic protocols [133,134].

Based on our review, pharmaceutical methods of terminating pregnancies are more advantageous than currently practiced physical methods in NHPs. Some drug interventions are characterized by lower invasiveness (e.g., cloprostenol), while others allow for protocol adjustment according to species-specific considerations and gestational stage (e.g., mifepristone combined with misoprostol). Some methods discussed in this review, such as methotrexate and anti-placental IgG, were successful at terminating pregnancy but had concerning side effects. Further research should be done on these drugs to determine the safest and most efficacious dose and route of administration before routine clinical use is considered. PGs and antiprogestins still represent the best approach for inducing abortion, especially if combined. PGs alone are often insufficient to reliably terminate pregnancy, most likely because of their limited effect in blocking progesterone. Cloprostenol is an exception in that it terminates pregnancy with high efficacy in single-drug protocols in marmosets. Thus, due to its efficacy and safety, cloprostenol might be considered the best option for marmosets. However, there is no evidence about its application in other NHP species [75,76]. In macaques, combination treatments provided the highest efficacy and safety. In particular, the combination of mifepristone with misoprostol is supported by the most extensive and robust data [3,96]. Other approaches, such as dinoprostone or carboprost vaginal rings, might provide interesting alternatives but require more extensive validation.

## Figures and Tables

**Table 1:** Overview of the pharmaceutical methods described in the literature that are used to terminate pregnancy in non-human primates.

Section		
4.1	Prostaglandins and combinations	
4.1.1		Misoprostol
4.1.2		Cloprostenol
4.1.3		Dinoprostone
4.1.4		Carboprost
4.2	Antiprogestins	
4.2.1		Mifepristone (RU-486)
4.2.2		Mifepristone + misoprostol
4.2.3		Lilopristone (ZK 98.734)
4.3	Methotrexate	
4.4	Heterologous placental immunoglobulins	

## Data Availability

Data are available on request.
